# Origin of the low thermal isomerization rate of rhodopsin chromophore

**DOI:** 10.1038/srep11081

**Published:** 2015-06-10

**Authors:** Masataka Yanagawa, Keiichi Kojima, Takahiro Yamashita, Yasushi Imamoto, Take Matsuyama, Koji Nakanishi, Yumiko Yamano, Akimori Wada, Yasushi Sako, Yoshinori Shichida

**Affiliations:** 1 Cellular Informatics Laboratory, RIKEN, 2-1 Hirosawa, Wako 351-0198, Japan; 2 Department of Biophysics, Graduate School of Science, Kyoto University, Kyoto 606-8502, Japan; 3 Department of Chemistry, Columbia University, New York, NY 10027, USA; 4 Department of Organic Chemistry for Life Science, Kobe Pharmaceutical University, Kobe 658-8558, Japan

## Abstract

Low dark noise is a prerequisite for rod cells, which mediate our dim-light vision. The low dark noise is achieved by the extremely stable character of the rod visual pigment, rhodopsin, which evolved from less stable cone visual pigments. We have developed a biochemical method to quickly evaluate the thermal activation rate of visual pigments. Using an isomerization locked chromophore, we confirmed that thermal isomerization of the chromophore is the sole cause of thermal activation. Interestingly, we revealed an unexpected correlation between the thermal stability of the dark state and that of the active intermediate MetaII. Furthermore, we assessed key residues in rhodopsin and cone visual pigments by mutation analysis and identified two critical residues (E122 and I189) in the retinal binding pocket which account for the extremely low thermal activation rate of rhodopsin.

## Introduction

Vertebrate eyes utilize two types of photoreceptor cells for dim- and bright-light conditions. Rod photoreceptor cells mediate dim-light vision, whereas cone photoreceptor cells drive vision under bright light. This division of labor between rods and cones allows our eyes to cover a wide dynamic range of detection, covering 11 orders of magnitude of light intensity^1^. Rods contain tens of millions of the photoreceptive molecule, rhodopsin, allowing it to respond to even a single photon^2^. Rhodopsin is a light-sensitive G protein-coupled receptor whose G protein activity is regulated by cis-trans photoisomerization of the retinal ligand. A single photon triggering the photoisomerization of a single rhodopsin molecule can result in a rod response. A prominent feature of rhodopsin is that, in the absence of light, it is extremely stable. The extremely low thermal activation rate of rhodopsin in the absence of light is essential for the function of rods as dim-light photoreceptors, because increased thermal activation, known as dark noise, would mask light triggered events and therefore increase the threshold of detection. In spite of the large amount of rhodopsin present in rods, a dark event (thermal activation) is only encountered a few minutes apart, which makes it extremely rare.

The thermal activation of rhodopsin was originally detected by electrophysiological experiments as discrete noise of dark-adapted rods^3^. Recordings of rod outer segment photocurrents of the transgenic mice’s rods containing red- or green-sensitive cone pigments indicate that rhodopsin’s isomerization rate is 1000 times lower in comparison with cone visual pigments^4^,^5^. Phylogenetic analyses have shown that cone pigments are ancestral to rhodopsin, indicating that rhodopsin emerged from cone pigments^6^. Therefore, suppression of the visual pigment dark noise must have been a critical step in the evolution of visual pigments to generate rods capable of responding to single photons.

Differences in the thermal activation rate (k_th_) between rhodopsin and cone pigments originate from differences in their amino acid sequences. As rhodopsin and cone visual pigments have similar amino acid sequences, the amino acid residues responsible for the low k_th_ of rhodopsin can be elucidated by mutational analysis, which targets key sites differing between rhodopsin and cone pigments. Until now, electrophysiology was the only experimental approach to measure the k_th_ of visual pigments. However, it is unrealistic to generate multiple knock-in animals whose rhodopsin is replaced by a mutant of rhodopsin or cone pigment and carry out electrophysiological measurements. Therefore, here we developed a biochemical method employing a non-isomerizable retinal analog, 11-cis-locked-7-membered-ring-retinal^7^, to compare the k_th_ ratio of visual pigments purified from cultured cells. Our mutational analysis revealed two amino acid residues required for the high thermal stability of rhodopsin.

## Results and Discussion

### Thermal activation of visual pigments originates exclusively from thermal *cis-trans* isomerization of the retinal chromophore

**Figure 1:**
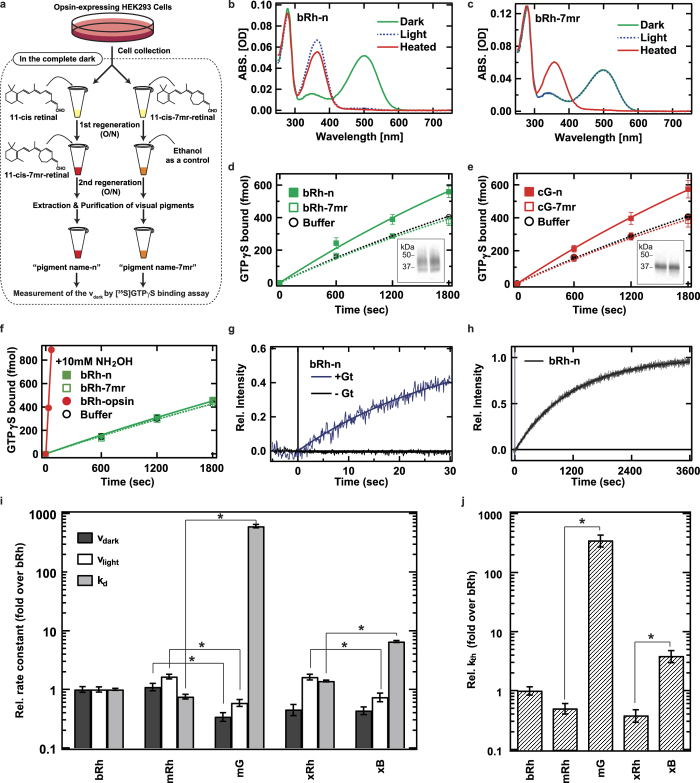
Comparison of v_dark_, v_light_, k_d_ and k_th_ of wild-type visual pigments (**a**) Schematic procedure of the sample preparation for the measurement of k_th_. (**b**,**c**) Absolute spectra of bRh-n and bRh-7mr in DM with 100 mM NH_2_OH. Dark, light-irradiated, and heat-denatured samples are shown by green, blue-dashed, and red lines, respectively. (**d**,**e**) G protein activation rate in the complete dark by bRh-n (green closed square), bRh-7mr (green open square, n = 5), cG-n (red closed square), cG-7mr (red open square) and buffer (black open circle). The western blotting data were cropped and shown in the inset (left lane: bRh-n or cG-n, right lane: bRh-7mr or cG-7mr). (**f**) G protein activation rate in the complete dark with 10 mM NH_2_OH by bRh-n (green closed square), bRh-7mr (green open square), bRh-opsin (red closed square), and buffer (black open circle). The initial rate of G protein activation by bRh-opsin was estimated from the linear fitting (red line) to be 126-fold higher than v_dark_ of bRh-n in (d). (**g**) Measurements of v_light_ and k_d_ of bRh-n by monitoring the change of intrinsic tryptophan fluorescence after flash light irradiation with (blue line) or without (black line) Gt. Intensities were normalized to the fluorescence increase of Gt in the presence of aluminium fluoride (**h**) Measurement of k_d_ of bRh-n by change of intrinsic tryptophan fluorescence after light irradiation. (**i**) Comparison of the v_dark_, v_light,_ and k_d_ of visual pigments measured by the same methods as shown in (d, g, h). (**j**) Comparison of the k_th_ of visual pigments estimated from data in (i). * indicates a significant difference of relative rate constants between visual pigments connected with a line (p < 0.05; Student’s t- test, two-tailed). All error bars in Fig. 1 represent the S.E.M. of more than three independent measurements.

**Figure 2:**
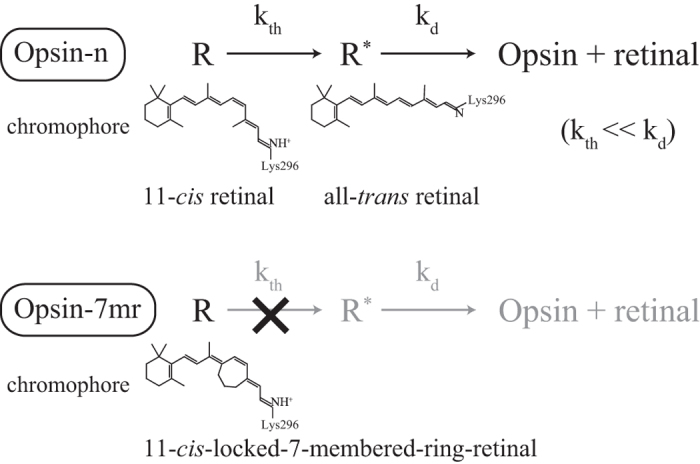
Two-step reaction scheme of thermal activation and deactivation of visual pigments. R and R* indicate visual pigments in the inactive and active state, respectively. The k_th_ indicates a thermal activation rate constant of R. The k_d_ indicates a thermal decay rate constant of R*. An opsin regenerated by normal 11*-cis* retinal (Opsin-n) spontaneously becomes R^*^ by thermal *cis-trans* isomerization of retinal in the complete darkness. After the first reaction, R* is degraded into opsin and retinal. In contrast, an opsin regenerated by 11-*cis*-locked-7-membered-ring-retinal (Opsin-7mr) cannot become R* due to the inhibition of *cis-trans* isomerization.

We first investigated whether or not thermal activation of rhodopsin and cone pigments really originates from the thermal *cis*-*trans* isomerization of their chromophores. The possibility that thermal activation is achieved without *cis*-*trans* isomerization arises in the framework of the two-state model, where the receptor fluctuates between active and inactive states, even in the presence of an inverse agonist such as 11-*cis*-retinal. In order to discriminate thermal isomerization of the chromophore from thermal fluctuations of the protein moiety, we used 11-*cis*-locked-7-membered-ring-retinal (7mr, Fig. 1a,2)^7^, a retinal analogue which is locked at 11-*cis* and cannot be isomerized to all-*trans*. Previous spectroscopic studies demonstrated that the rhodopsin regenerated with 7mr cannot form a batho-intermediate and thus it is unbleachable, even after photon absorbtion^7^,^8^. Moreover, an electrophysiological study demonstrated that the addition of 7mr into a photobleached rod cell substantially restored the sensitivity from bleaching adaptation, which would be due to a suppression of a constitutive activity of retinal free opsin^9^.

Visual pigments were purified from pigment-expressing HEK293 cells as described in Methods. In the process of purification, the opsin-containing cell membrane was divided into two aliquots, and each aliquot was regenerated with an excessive amount of native 11-*cis*-retinal or 7mr. Then, the former aliquot was additionally regenerated with 7mr to remove the effect of an affinity difference between 11-*cis*-retinal and 7mr, if any. (Fig. 1a). (The former aliquot is abbreviated to “pigment name-n”, and the latter aliquot is to “pigment name-7mr”.) Fig. 1b,c show absorbance spectra of purified bovine rhodopsin (bRh). Almost all of the opsin in bRh-n was regenerated by 11-*cis*-retinal, which can be bleached by light (Fig. 1b). In contrast, the opsin in bRh-7mr was regenerated by 7mr, which cannot be bleached by light because the *cis-trans* isomerization of the chromophore is inhibited by the 7-membered-ring (Fig. 1a,c)^10^.

We next measured the G protein activation ability of the visual pigments by a [^35^S]GTPγS binding assay in complete darkness (under infrared light). The [^35^S]GTPγS binding assay is a commonly used technique to evaluate the G protein activation rate of G protein-coupled receptors by measuring the GDP-GTPγS exchange rate of G proteins *in vitro*^11^ (See Methods). The concentrations of visual pigments in the aliquots were compared by Western blotting analysis (Fig. 1d,e, Supplementary Fig. 2, inset). No significant elevation of Gt activation over the intrinsic GDP-GTPγS exchange rate was observed in the presence of bRh-7mr (Fig. 1d, open squares). In contrast, a small but significant elevation of Gt activation rate was observed in the presence of bRh-n (Fig. 1d, closed squares). The similar results were observed in the analysis of chicken green-sensitive cone pigment (cG) as shown in Fig. 1e. These results clearly show that thermal isomerization of the chromophore is the sole cause of light-independent generation of the activation state, both in rhodopsin and cone pigment.

We excluded the possibility that the Gt activation was derived from accumulated opsin apoprotein, by measuring the catalytic activity of bRh-n upon addition of 10 mM NH_2_OH in the dark. The NH_2_OH accelerates the depletion of all-trans retinal from visual pigments in MetaII state, as it reacts with retinal and forms retinal oxime. The catalytic activity of bRh-n was radically suppressed by NH_2_OH as result of the accelerated MetaII decay, whereas the opsin (photobleached bRh-n) showed significant catalytic activity under the same condition (Fig. 1f). We can therefore conclude that the catalytic activity of bRh-n without NH_2_OH in the dark is exclusively derived from the MetaII state generated by thermal isomerization of its 11-*cis*-retinal chromophore (Fig. 1d).

Previous electrophysiological studies have already suggested that the discrete noise of dark current originates from thermal isomerization of single rhodopsin molecules, based primarily on the observation that discrete noise and single photon response of rod cells are virtually identical^12^. Moreover, the significant difference in the frequency of discrete noise between A1- and A2-11-*cis* retinal containing rods further supported this notion^13^,^14^. The present biochemical analysis directly confirms this view by observing the inhibitory effect of non-isomeraizable retinal analog (7-membered retinal) to the generation of dark noise. Therefore, the *cis-trans* isomerization of the chromophore is essential for the dark noise.

### Comparison of k_th_ of visual pigments

We next developed a theoretical framework to quantitatively evaluate k_th_ across different pigments. First, we assumed a simplified two-step reaction scheme as shown in Fig. 2, where R is the pigment in the inactive state and R* is the thermally activated pigment. Given that the thermal activation of R occurs much slower than the decay of R* (k_th_ << k_d_), a steady state approximation can be applied to the concentration of R*. (Here, k_d_ is the spontaneous decay rate of activated pigment without interaction with rhodopsin kinase and arrestin.) Therefore we obtain the following equation 1:


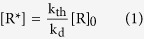


where [R]_0_ is the initial concentration of the pigment. Namely, it can be considered that a small but constant concentration of R* exists in a solution of the purified pigments in the dark. Because R* is essentially the same as the light-induced MetaII state^3^, the initial rate of G protein activation by a pigment in the dark (v_dark_) can be approximated by equation 2:


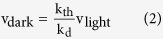


where v_light_ is the initial rate of G protein activation by a MetaII generated by light. Therefore, we can estimate the k_th_ from measurements of v_dark_, v_light_, and k_d_ as equation 3:


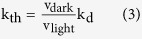


To validate the applicability of this method, we first compared the k_th_ of mouse rhodopsin (mRh), mouse green-sensitive cone pigment (mG), xenopus rhodopsin (xRh), and xenopus blue-sensitive cone pigment (xB), which have previously been characterized by several electrophysiological studies^5^,^15^,^16^.

As shown above, we estimated v_dark_ by comparing dark activities of pigments containing native 11-*cis*-retinal and 7mr. Measurement of v_light_ and k_d_ was carried out with a photon counting system as previously reported^17^,^18^, by measuring the intrinsic tryptophan fluorescence emission changes of Gt^19^ and visual pigments^20^. The v_light_ of bRh was estimated from the difference of initial rates of light-dependent fluorescence increase of bRh-n with and without Gt (Fig. 1g). The k_d_ of bRh was estimated from the rate constant of the fluorescence increase of bRh-n without Gt (Fig. 1h). We also confirmed that the k_d_ of bRh-n estimated from the fluorescence increase is identical to those estimated from the [^35^S]GTPγS binding assay (Supplementary Fig. 1). Similarly, we estimated and compared the v_dark_, v_light_, and k_d_ of other visual pigments (Fig. 1i and Supplementary Fig. 2,3,4).

Finally, we estimated the ratio of the k_th_ of visual pigments based on the results of these measurements and equation 3. The v_dark_ and v_light_ of mG were 0.31- and 0.35-fold those of mRh, respectively, in contrast with the k_d_ that was 810-fold (Fig. 1i). Thus, the k_th_ of mG was estimated to be 710-fold that of mRh (Fig. 1j). This ratio is consistent with the previous electrophysiological analysis of mG knock-in mouse^5^. On the other hand, the k_th_ of xB was estimated to be 10.1-fold that of xRh (Fig. 1j), with v_dark_, v_light_, and k_d_ of xB estimated at about 1.0, 0.45, and 4.7-fold those of xRh, respectively (Fig. 1i). Two different k_th_ values of the blue visual pigment have been previously reported for green rods of toads, by electrophysiological studies. The earlier study reported that the k_th_ of the blue pigment in green rods is 4-fold that of rhodopsin in red rods^16^, whereas, a more recent study reported it to be 0.022-fold^15^. While the reason for the 180-fold discrepancy is unclear, our analysis is consistent with the earlier study^16^.

### Key amino acid residues for the extraordinary low k_th_ of rhodopsin

**Figure 3:**
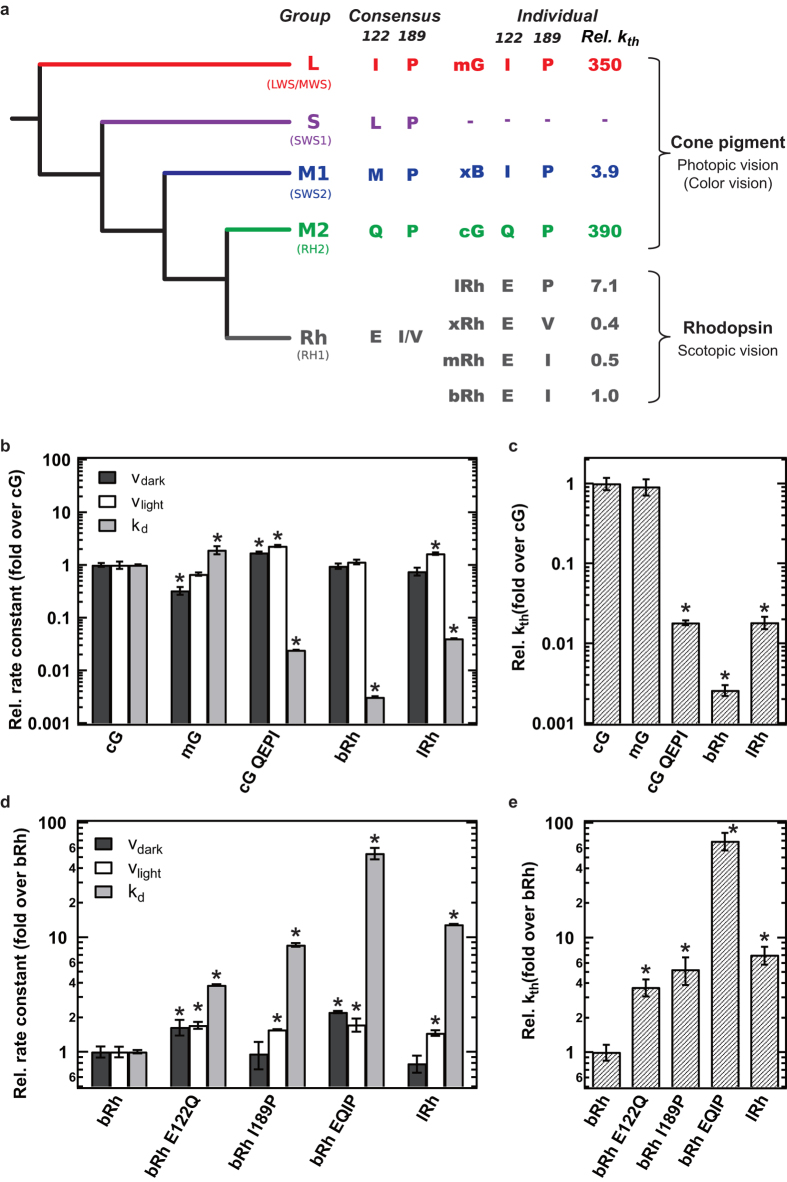
Comparison of the k_th_ of the wild-type and mutants of bRh, cG, and lRh (**a**) Schematic diagram of phylogenetic relationship of vertebrate visual pigments. The amino acids at position 122 and 189 conserved in each group (consensus) and those of each visual pigment analyzed in the present study (individual) are denoted. The right column (Rel. k_th_) shows the relative k_th_ of visual pigments estimated from the analysis shown in the text (fold over bRh). The complete phylogenetic tree is available as Supplementary Fig. 5. (**b**-**e**) The v_dark_, v_light,_ k_d_ of the wild-type and mutants of visual pigments were compared in (b,d). The k_th_ estimated from the data in (b) and (d) were compared in (c) and (e), respectively. Error bars represent the S.E.M. of more than three independent measurements. * indicates a significant difference of relative rate constants between visual pigments relative to cG in (b,c) and bRh in (d,e) (p < 0.05; Student’s t- test, two-tailed).

In comparison with v_dark_ and v_light_, k_d_ varied substantially between mRh and mG (Fig. 1i), suggesting that the k_th_ is highly correlated with the k_d_. To test this hypothesis, we compared the k_th_ of wild-type and mutants of cG and bRh. Previous studies have shown that the two residues E122 and I189 are responsible for the low k_d_ of rhodopsin relative to cG^21^,^22^. Phylogenetically, vertebrate visual pigments are classified into 5 groups based on their primary sequence analysis^6^,^23^ (Fig. 3a, Supplementary Fig. 5). Rhodopsin belongs to RH (RH1) group, which is a sister group to M2 (RH2) group containing cG. On the other hand, the mG belongs to L (LWS/MWS) group, the most distant group to RH group. Thus, the comparison between rhodopsin and cG and their mutants should provide us an insight on the acquisition of the low k_th_ of rhodopsin in the molecular evolutionary process.

Fig. 3b shows that the k_d_ of cG is 320-fold that of bRh, in contrast with the relatively similar values of v_dark_ or v_light_. The k_th_ of cG was estimated to be 390-fold that of bRh and to be comparable to that of mG (Fig. 3c). Moreover, the double mutation Q122E/P189I of cG (cG QEPI) caused slight increase of the v_dark_ and v_light_ (1.7- and 2.3-fold, respectively) and substantial decrease of k_d_ (0.024-fold) (Fig. 3b). The k_th_ of cG QEPI was estimated to be 0.018-fold that of wild-type cG, indicating that the double mutation enhanced the thermal stability of both inactive and active states of cG (Fig. 3b,c). It should be noted that the k_th_ of cG QEPI was still 7-fold that of bRh (Fig. 3c), indicating that other residues may also contribute to the low k_th_ of rhodopsin. On the other hand, the double mutation E122Q/I189P of bRh (bRh EQIP) resulted in about 70-fold increase of k_th_ (Fig. 3e), considering that the v_dark_, v_light_, and k_d_ were increased by a factor of 2.2, 1.7, and 54, respectively (Fig. 3d). We also performed single mutation analyses of bRh (E122Q and I189P), to further elucidate their roles. Based on the measured v_dark_, v_light_, and k_d_ of the single mutants (Fig. 3f), only a small increase (4 ~ 5-fold) of k_th_ can be attributed to each mutation (Fig. 3e).

Lamprey rhodopsin (lRh) has an intermediate amino acid sequence between mammalian rhodopsin and cone pigment; E122 and P189, and it can be considered as an intermediate form between rhodopsin and cone pigments (Fig. 3a, Supplementary Fig. 5)^24^. We analyzed the k_th_ of lRh to test whether or not agnathans have acquired the characteristic higher thermal stability of the inactive conformation observed in rhodopsin. As expected, the k_th_ of lRh showed an intermediate value, consistent with the analysis of bRh I189P; 0.018-fold that of cG and 7-fold that of bRh (Fig. 3c,e). As agnatha sits at the base of vertebrate animals, these results suggest that the acquisition of other important residues, including I189, further enhanced the thermal stability of rhodopsin at a later stage in the evolutionary process.

### A link between thermal isomerization and active state lifetime

**Figure 4:**
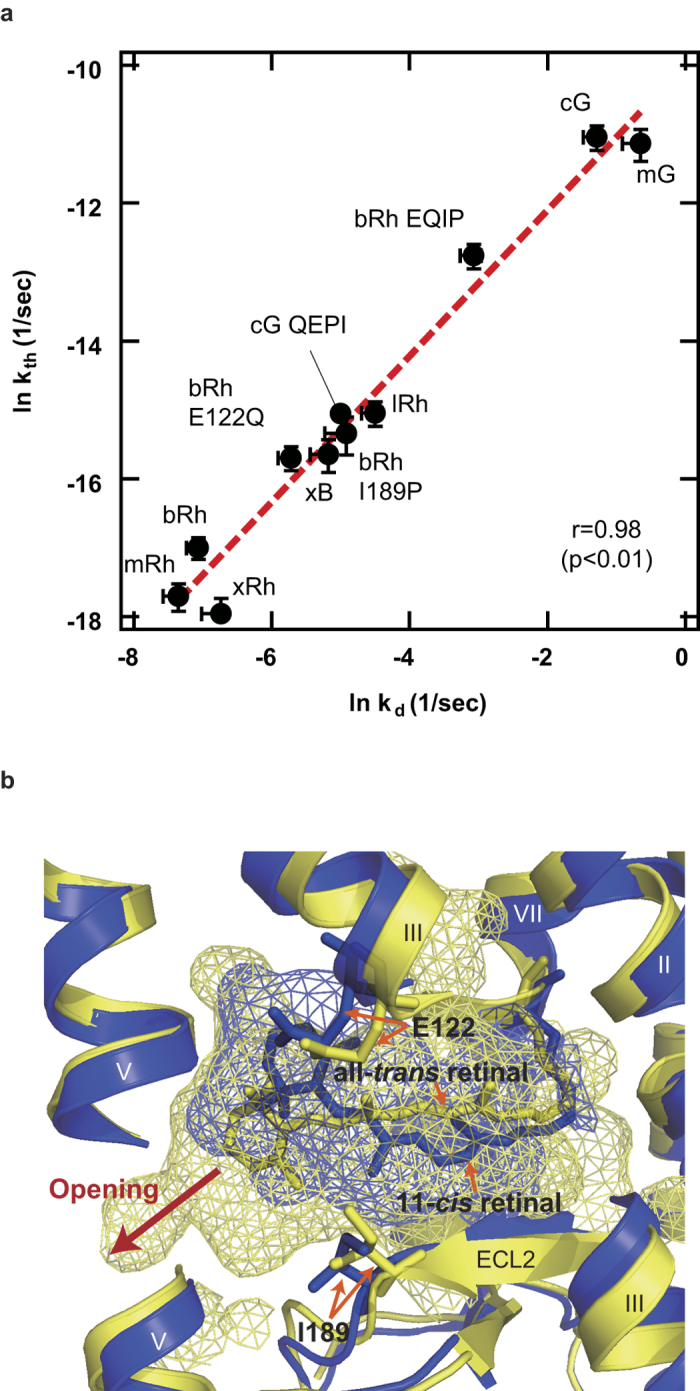
Correlation between the k_th_ and k_d_, and comparison of the retinal binding pocket between inactive and active states of bRh (**a**) Correlation between ln k_th_ and ln k_d_ of visual pigments. Error bars represent the S.E.M. of more than three independent measurements. The regression line is shown by a red-dashed line; ln k_th_ = ln k_d_ - 10. (**b**) The crystal structures of dark (blue: 1U19^32^) and Meta II (yellow: 3PXO^33^) states of bRh. The cavities around the RBP were shown in mesh. In the crystal structure of Meta II state, the RBP is open for the retinal release (red arrow) in contrast with the tightly closed RBP in the dark state. The E122 and I189 constitute the RBP both in the dark and Meta II states. The crystal structures were depicted using PyMol (http://www.pymol.org/)^36^.

The results obtained in the present study indicate a strong correlation between k_th_ and k_d_ (Fig. 4a). The extremely low k_d_ is a well-known characteristic that distinguish rhodopsins from cone pigments^6^. However, the physiological significance of the excessively long-lasting active intermediate of rhodopsin (>min) has been unclear, because MetaII is deactivated by rhodopsin kinase and arrestin immediately after its formation *in vivo* (~100 ms)^25^. The clear correlation between k_th_ and k_d_ indicates that the thermal isomerization and dissociation rates of the chromophore are indivisible factors of visual pigments (Fig. 4a). Thus, it makes more sense to think of the excessively low k_d_ of rhodopsin as a byproduct of the extremely low dark noise.

The correlation between the thermal activation rate and the active state lifetime could also indicate that the thermal stability of visual pigment is a trade-off between the detection threshold and the adaptation speed of photoreceptor cells. Although G protein signaling is quickly stopped by arrestin, a key processes of recovery from bleaching desensitization is the release of all-trans retinal and re-uptake of 11-cis retinal^26^. Assuming that the photoactivated rhodopsin cannot be regenerated during the long lasting active state, rods would be forced to prolong the time of dark adaptation to suppress the dark noise. On the other hand, cones would be able to quickly recover from bleaching desensitization, at the cost of elevated dark noise.

### Molecular mechanism of low thermal isomerization rate

A previous theoretical study suggested that an electrostatic interaction in the salt bridge between retinal Schiff base and opsin moiety enhances the energy barrier to thermal isomerization of retinal in rhodopsin compared with that in solution^27^,^28^. More recently, an electrophysiological study has shown a quantitative relationship between activation energy (E_a_) and absorption maximum (λ_max_)^15^, supporting the long-standing Barlow’s hypothesis that λ_max_ and the thermal activation rate (k_th_) of visual pigments are related^29^. This was also emphasized from a theoretical calculation^30^. However, as described earlier, the λ_max_ effect alone cannot by itself account for the difference in k_th_ between rhodopsin and cone pigments. In fact, the predicted k_th_ ratio between rhodopsin and cone pigments was 15 ~ 120-fold different from the measured values in Luo *et al*^15^ and even 4-orders of magnitude different in Ala-Laurila *et al*^31^, if only the effect of λ_max_ is considered. Moreover, mG showed the k_th_ that is 860-fold than that of mRh^5^, whereas only a two-fold increase at most is expected from the 8 nm λ_max_ difference between mRh and mG^15^.

The rate of thermal isomerization can be expressed as 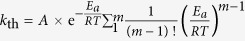 based on the Hinshelwood distribution^31^ (*R* is the gas constant, *T* is absolute temperature, and *m* is the number of molecular vibrational modes contributing thermal energy to pigment activation), where *E*_a_ is inversely proportional to the pigment’s λ_max_^15^. Assuming that the proportionality constant between *E*_a_ and photon energy at λ_max_ and *m* do not largely differ between rhodopsin and cone pigments, our results indicate that the pre-exponential factor (*A*) of the green-sensitive cone pigment is two orders of magnitude greater than that of rhodopsin. This is an intermediate value between those estimated by Luo *et al* and by Ala-Laurila *et al*^31^. Using the Eyring equation, the 100-fold increase of A (and therefore k_th_) can be accounted for by a 4.6-fold ΔS^‡^ for the thermal isomerization. The difference in A could also be explained by assuming an equilibrium between two substates. As the chromophore is tightly fixed in the retinal binding pocket (RBP)^32^, we hypothesize that the RBP transiently opens for the *cis*-*trans* isomerization like the MetaII state^33^ (Fig. 4b). A pigment, therefore, can be thought to exist in equilibrium (*K*_*p*_ = [P*]/[P]), where one of the substates (P*) has an opened RBP and facilitates the thermal isomerization^34^. Our results suggest that the *K*_*p*_ of cone pigments is two orders of magnitude greater than that of rhodopsin. This notion is in line with the greater k_d_ values of cone pigments compared to rhodopsins, as k_d_ is affected by accessibility of water molecules to the RBP in the active state of visual pigments^35^. The strong correlation between k_th_ and k_d_ suggests that the factors that determine the population of opened RBP in the dark state, probably interactions involving the residues at positions 122 and 189 (Fig. 4b), are also present in the active state.

In closing, we have developed a biochemical protocol for the measurement of k_th_, which paves the way to analyze mutants of visual pigments without making a transgenic animal. Our biochemical approach allowed us to directly show that thermal activation of pigments in the dark originates exclusively from thermal isomerization of the chromophore. The lead-time for measuring k_th_ was shortened from years to weeks, and we have finally revealed that during the course of molecular evolution, acquisition of E122 and I189 suppressed the thermal fluctuation of RBP in rhodopsin, resulting in the extraordinary low dark noise of rod photoreceptor cells.

## Methods

## Additional Information

**How to cite this article**: Yanagawa, M. *et al*. Origin of the low thermal isomerization rate of rhodopsin chromophore. *Sci. Rep*. **5**, 11081; doi: 10.1038/srep11081 (2015).

## Supplementary Material

Supplementary Information
