# Supplementary material for: Origin of the low thermal isomerization rate of rhodopsin chromophore
Source: Sci Rep. 2015 Jun 10;5:11081. doi: 10.1038/srep11081 (PMC4462023; doi:10.1038/srep11081)

Supplementary Information

Title: Origin of the low thermal isomerization rate of rhodopsin chromophore

Authors: Masataka Yanagawa[1, 5], Keiichi Kojima[2, 5], Takahiro Yamashita[2], Yasushi Imamoto[2], Take Matsuyama[2], Koji Nakanishi[3], Yumiko Yamano[4], Akimori Wada[4], Yasushi Sako[1], and Yoshinori Shichida[2*]

Affiliations and footnotes:

[1] Cellular Informatics Laboratory, RIKEN, 2-1 Hirosawa, Wako 351-0198, Japan.

[2] Department of Biophysics, Graduate School of Science, Kyoto University, Kyoto 606-8502, Japan.

[3] Department of Chemistry, Columbia University, New York, NY 10027, USA

[4] Department of Organic Chemistry for Life Science, Kobe Pharmaceutical University, Kobe 658-8558, Japan

[5] These authors contributed equally to this work.

*e-mail: shichida@rh.biophys.kyoto-u.ac.jp

Corresponding Author: Yoshinori Shichida,

Department of Biophysics, Graduate School of Science, Kyoto University, Kyoto 606-8502, Japan.

Phone: +81-75-753-4213 Email: shichida@rh.biophys.kyoto-u.ac.jp

Contents:

Supplementary Methods

Supplementary References 37-40

Supplementary Figure Legends 1-5

Supplementary Figures 1-5

Supplementary Methods

Materials

[^{35}S]guanosine 5'-3-O-(thio)triphosphate (GTPγS) (37 TBq/mmol) was purchased from PerkinElmer Life Sciences. 11-*cis*-locked-7-membered retinal (7mr) was synthesized as previously described[7].

Heterologous expression and purification of visual pigments

The cDNA of visual pigments were tagged with the epitope sequence of the anti-bovine rhodopsin monoclonal antibody Rho1D4 at the C-terminus for purification. Site directed mutagenesis was performed by using QuikChange kit (Agilent Technologies). The wild-type and mutant cDNAs of the pigments were introduced into the mammalian expression vector pcDNA3.1 (Life Technologies), pUSRα[37] or pCAGGS[38]. Expression of visual pigments in HEK293 cells was performed by the methods previously reported[11]. HEK293 cells were grown to ~ 40 % confluency in DMEM/F12 supplemented with 10 % fetal bovine serum and were transfected with wild-type or mutant pigments plasmid DNA (10 μg / 100-mm dish) using the calcium-phosphate method. The cells were collected by centrifugation 48 hr after transfection and divided into two aliquots (Fig. 1a). One aliquot was incubated with an excess concentration of 11-*cis*-retinal (500 μM), and the other aliquot was with 7mr (500 μM). After 24 hr incubation at 4 °C, the former aliquot was additionally incubated with 7mr (500 μM), and the latter aliquot was with ethanol as a mock. After another 24 hr incubation, the pigments was extracted by using 1% n-dodecyl β-D-maltoside (DM) in buffer A, and the DM extract was incubated with Rho1D4 antibody-agarose for 3 hr ~ overnight at 4 °C. After washing with 0.02% DM in buffer A, rhodopsin was eluted with the same buffer containing the C-terminal nonapeptide of bovine rhodopsin. All the experiments after incubation of retinal were performed in the complete darkness with an infrared night vision device. The quantification of the purified pigment was

performed by UV-vis spectrophotometry using UV-2400 (Shimadzu). The visual pigment regenerated by 7mr, which cannot be bleached by light, was denatured by heating (70 °C, 3 min) in the presence of 10 ~ 100 mM NH_2OH. The bRh-opsin in Fig. 1f is made from bRh-n by complete photobleaching in the presence of 10 mM NH_2OH.

Western blotting

Western blotting analysis of purified visual pigments was performed as previously described[39]. The purified pigments in the two aliquots were diluted in the same ratio and suspended in the buffer [62.5 mM tris(hydroxymethyl)aminomethane (pH 6.8), 4% SDS, 10% glycerol, 2.5% β-mercaptoethanol]. After the SDS-PAGE, the electrophoresed proteins were transferred onto a polyvinylidene difluoride membrane and probed with the Rho1D4 antibody. Immunoreactive proteins were detected by the ABC method and were visualized using the horseradish peroxidase-diaminobenzidine reaction.

Measurement of v_{dark}

The v_{dark} was measured by [^{35}S]GTPγS binding assay in the complete darkness with a night vision device. The purified pigment or 0.02% DM/buffer A as a mock was preincubated with [^{35}S]GTPγS solution. After 10 min incubation at 20 °C, the GDP/GTPγS exchange reaction was started by adding Gt solution purified from bovine retina. The assay mixture (40 μl) consisted of 300 nM pigment (or opsin), 1 μM Gt, 5 μM GTPγS, 25 nM [^{35}S]GTPγS, 0.015% DM, 50 mM HEPES (pH 6.5), 140 mM NaCl, 5.8 mM $MgCl_2$, and 1 mM DTT. After incubation for 0 (immediately after mixture), 10, 20, and 30 min, the 5 μl of assay mixture was added into the stop solution [200 μl: 5 μM GTPγS in buffer C (20 mM tris(hydroxymethyl)aminomethane (pH 7.4), 100 mM NaCl, 25 mM $MgCl_2$)] to terminate the GDP/[^{35}S]GTPγS exchange reaction. Then, the sample in the stop solution was immediately filtered through a nitrocellulose membrane to trap [^{35}S]GTPγS bound to G proteins, and after that, the membrane was

immediately washed 3 times with buffer C to remove the nonspecific bound [^{35}S]GTPγS. The amount of [^{35}S]GTPγS was quantitated by assaying the membrane filter with liquid scintillation counter. Experimental data were fitted with a single exponential function, and the v_{dark} was estimated to be the difference of initial rates between two aliquots as described above.

Measurement of v_{light}

The measurement of v_{light} was performed according to the previous report[18]. The assay temperature (20 °C) and the final concentration of reaction mixture was the same as that in the measurement of v_{dark} except for the concentration of pigments (20 nM pigment, 0 or 1 μM Gt, 5 μM GTPγS, 25 nM [^{35}S]GTPγS, 0.015% DM, 50 mM HEPES (pH 6.5), 140 mM NaCl, 5.8 mM $MgCl_2$, and 1 mM DTT). Experimental data were fitted with a single exponential function, and the v_{light} was estimated as previously described[18].

Measurement of k_d

The measurement of k_d was performed according to the previous reports[17]. The assay temperature (20 °C) and the final concentration of reaction mixture was the same as that in the measurement of v_{light} without Gt (20 nM pigment (60 nM for cG and mG to increase the signal to noise ratio), 5 μM GTPγS, 25 nM [^{35}S]GTPγS, 0.015% DM, 50 mM HEPES (pH 6.5), 140 mM NaCl, 5.8 mM $MgCl_2$, and 1 mM DTT). Experimental data corresponding to the decay of MetaII were fitted with a single exponential function to estimate k_d.

Estimation of the k_d of bRh by the [^{35}S]GTPγS binding assay

To confirm that the k_d estimated from the fluorescence measurement is really reflecting the decay rate of R* in the scheme in Fig. 2, we also estimated the k_d of bRh by [^{35}S]GTPγS binding assay. The final concentration of reaction mixture (40 μl) was the same as the measurement of v_{light}. The GDP/GTPγS exchange reaction was

started by adding Gt solution after time intervals (30, 300, 900, 1800, 3600 sec) after light irradiation of bRh. After incubation for 30, 60, and 90 sec, the 5 μl of assay mixture was added into the stop solution and filtered through a nitrocellulose membrane. The protocol after filtration is the same as measurement of v_{dark}. The Initial rate of G protein activation ability of bRh in each condition was estimated from the fitted with a single exponential function. Because the photoactivated bRh was decayed during the intervals before addition of Gt solution according to the scheme in Fig. 2, the time course of the initial rates of G protein activation after light irradiation reflects the decay rate of R*. Then, the decay rate of R* was estimated from the fitting with a single exponential fitting function.

Supplementary References

37. Kayada, S., Hisatomi, O. & Tokunaga, F. Cloning and expression of frog rhodopsin cDNA. *Comp Biochem Physiol B Biochem Mol Biol* **110**, 599-604 (1995).
38. Niwa, H., Yamamura, K. & Miyazaki, J. Efficient selection for high-expression transfectants with a novel eukaryotic vector. *Gene* **108**, 193-9 (1991).
39. Yanagawa, M., Yamashita, T. & Shichida, Y. Comparative fluorescence resonance energy transfer analysis of metabotropic glutamate receptors: implications about the dimeric arrangement and rearrangement upon ligand bindings. *J Biol Chem* **286**, 22971-81 (2011).
40. Katoh, K., Kuma, K., Toh, H. & Miyata, T. MAFFT version 5: improvement in accuracy of multiple sequence alignment. *Nucleic Acids Res* **33**, 511-8 (2005).

Supplementary Figure Legends

Supplementary Fig. 1 Measurements of the v_{dark} by [^{35}S]GTPγS binding assay

The mRh-n (a), mG-n (b), xRh-n (c), xB-n (d), cG QEPI-n (e), lRh-n (f), bRh E122Q-n (g), bRh I189P-n (h), and bRh EQPI-n (i) were indicated by color-closed squares. The mRh-7mr (a), mG-7mr (b), xRh-7mr (c), xB-7mr (d), cG QEPI-7mr (e), lRh-7mr (f), bRh E122Q-7mr (g), bRh I189P-7mr (h), and bRh EQPI-7mr (i) were indicated by color-open squares. The buffer was indicated by black-open circle. Error bars represent the S.E.M of more than three independent measurements. The western blotting data were cropped and shown in the inset (left lane: aliquot regenerated by native 11-*cis*-retinal, right lane: aliquot regenerated by 7mr).

Supplementary Fig. 2 Comparison of the k_d of bRh estimated from [^{35}S]GTPγS binding assay and the fluorescence measurement

(a) Gt activation ability of photoactivated bRh-n after time intervals (30, 300, 900, 1800, 3600 sec) after light irradiation (closed circles). Intrinsic Gt activation was shown as Buffer (open circle). The initial rate of Gt activation ability was estimated from the single exponential fitting. (b) Time course of initial rates of Gt activation ability of bRh-n after light irradiation was plotted by green closed circle. The single exponential fitting of the time course was shown in green line. Blue line corresponds to the data shown in Fig. 1h, which were inverted and normalized to the green curve. Time constants estimated from the green fitting curve (1250 ± 80 sec) is good agreement with that estimated from the fluorescence increase (1110 ± 10 sec). Error bars represent the S.E.M of more than three independent measurements.

Supplementary Fig. 3 Measurement of v_{light} by monitoring the change of intrinsic tryptophan fluorescence after flash light irradiation

The change of intrinsic tryptophan fluorescence after flash light irradiation with or without Gt were indicated by blue or black line, respectively: (a) mRh-n, (b) mG-n, (c) xRh-n, (d) xB-n, (e) cG-n, (f) cG QEPI-n, (g) bRh E122Q-n, (h) bRh I189P-n, and (i) bRh EQPI-n, and (j) lRh-n. Intensities were normalized to the fluorescence increase in the presence of aluminium fluoride.

Supplementary Fig. 4 Measurement of k_d by monitoring the change of intrinsic tryptophan fluorescence after flash light irradiation

The change of intrinsic tryptophan fluorescence after light irradiation without Gt were indicated by black line: (a) mRh-n, (b) mG-n, (c) xRh-n, (d) xB-n, (e) cG-n, (f) cG QEPI-n, (g) bRh E122Q-n, (h) bRh I189P-n, and (i) bRh EQPI-n, and (j) lRh-n.

Supplementary Fig. 5 Phylogenetic analysis

Phylogenetic tree of vertebrate visual pigments. Nodes labels indicate consensus support (%) of 1000 bootstrap replicates. The tree shows 5 distinctive groups corresponding to 4 cone pigments and rhodopsin. Amino acid residues at 122 and 189 are shown on the right. Sequences of visual pigments from representative taxa were aligned by MAFFT[40] suing the G-INS-i option. Phylogenetic trees were then constructed by Neighbor Joining (NJ) as implemented by Geneious version 6, using default parameters. To cover a wide range of vertebrate species we chose species from representative taxa whose visual pigment sequences were available. The taxa represented in our data are: Petromyzontiformes (lamprey: AAR14681, AAR14680, AAR14682, AAR14683, AAR14684), Chondrichthyes (cartilaginous fishes: O93441, ABU84863,

NP_001279735, ABU84866, ABU84865, P79863, O93459, AFS63881), Actinopterygii (ray-finned fishes: XP_004070588, BAE78645, BAE78646, BAD99136, BAE78647, BAE78648, BAE78649, BAE78652, BAE78650, BAE78651, CAG11334, AAT38457, Q9DGG4, AAT38458, CAG06941, CAF96876, NP_571287, AAD24754, AAH76120, P35359, AAD24752, AAI64163, AAH75989, AAD24753, ADP06864, AAD24756, AAD24755), Actinistia (coelacanth: AAD30519, AAD30520), Dipnoi (lungfish: ABS89280, ABS89278, ABS89279, ABS89281, ABS89282), Anura (frogs & toads: NP_001096331, NP_001090803, NP_001119548, XP_002937272), Urodela (newts and salamanders: BAB55452, BAB79499, BAB39378, AAC96070, Q90245, AAC96071, AAC96069), Lacertilia (lizards: AAD25917, AAD25918, AAD45183, P35357, P35358, AAG61163, AAA17706, P41591, XP_003220394, AAD32621, XP_003216951, AAZ79907, AAZ79908, AAZ79909, AAZ79910), Serpentes (snakes: ACS49832, ACS49830, ACS49831, ACS49829, ACS49827, ACS49828), Aves (birds: CAA40727, NP_001025777, NP_990821, NP_990769, NP_990848, AAD38036, AAD32241, AAD32242, AAD38034, AAD38035), Chelonia (turtles & tortoises: ENSPSIG00000002239, XP_006119407, XP_006113270, XP_006132899, XP_005281338, XP_005309732, XP_005281339, XP_005288514, XP_005281346), Crocodylotarsi (crocodiles: XP_006269091, NP_001274211, XP_006269113), Monotrema (platypus and echidna: ACD85828, AFO7016, ACD85829, NP_001121097, NP_001121098), Marsupialia (marsupials: ACA28596, Q8HY69, AAR14685, NP_001138553, XP_001366225, NP_001138556) and Placentalia (placental mammals: NP_001014890, P03999, P04001, P04000, NP_000530, NP_663358, AAB53320, NP_032132). Additionally, the visual pigment of Ciona (NP_001027727) was included to root the tree.

Supplementary Figure 1

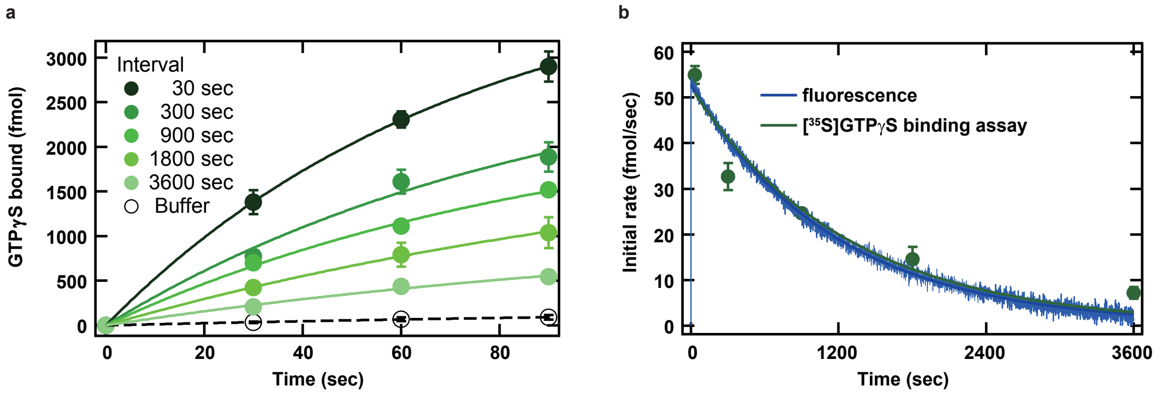

Supplementary Figure 2

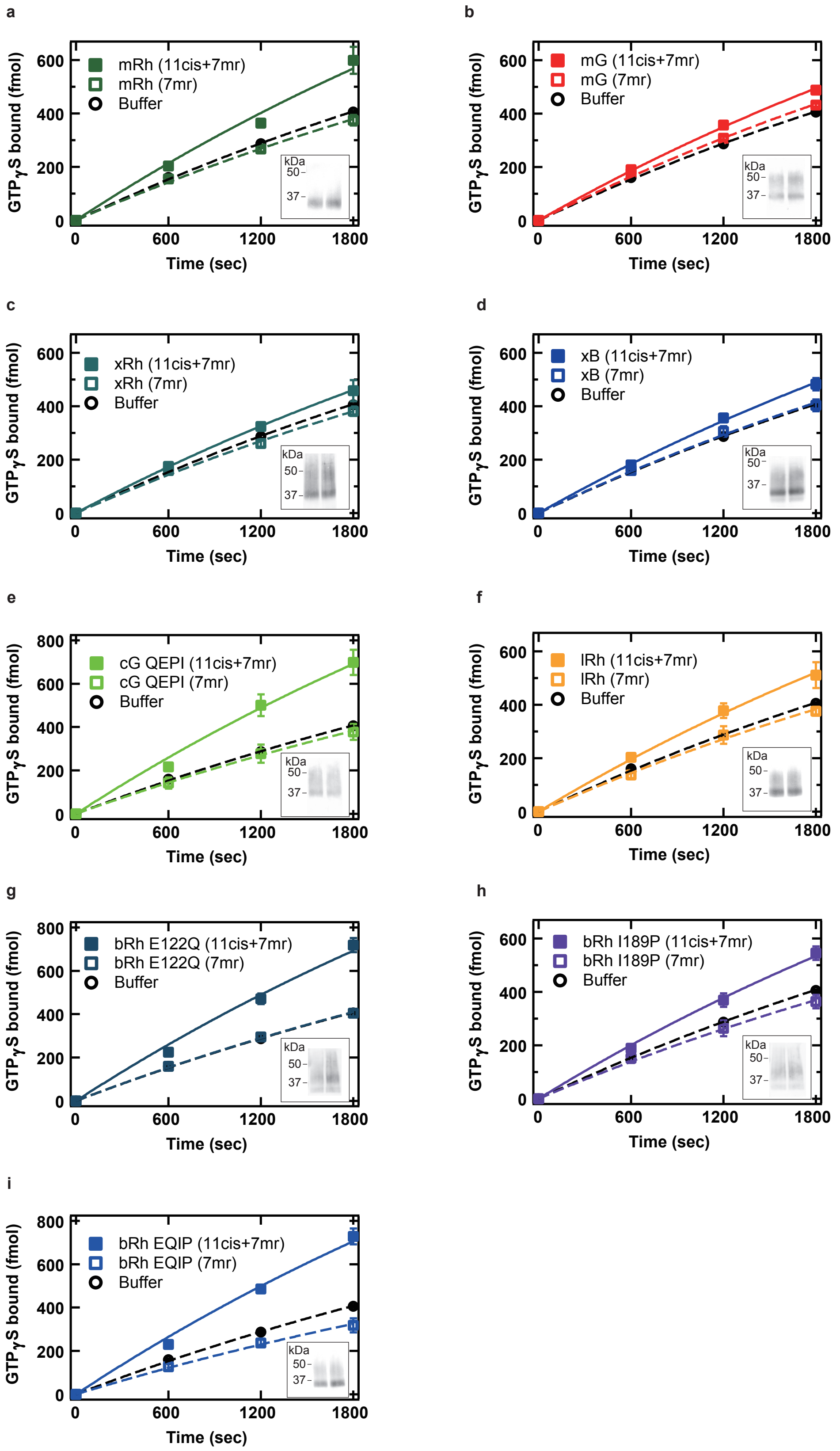

Supplementary Figure 3

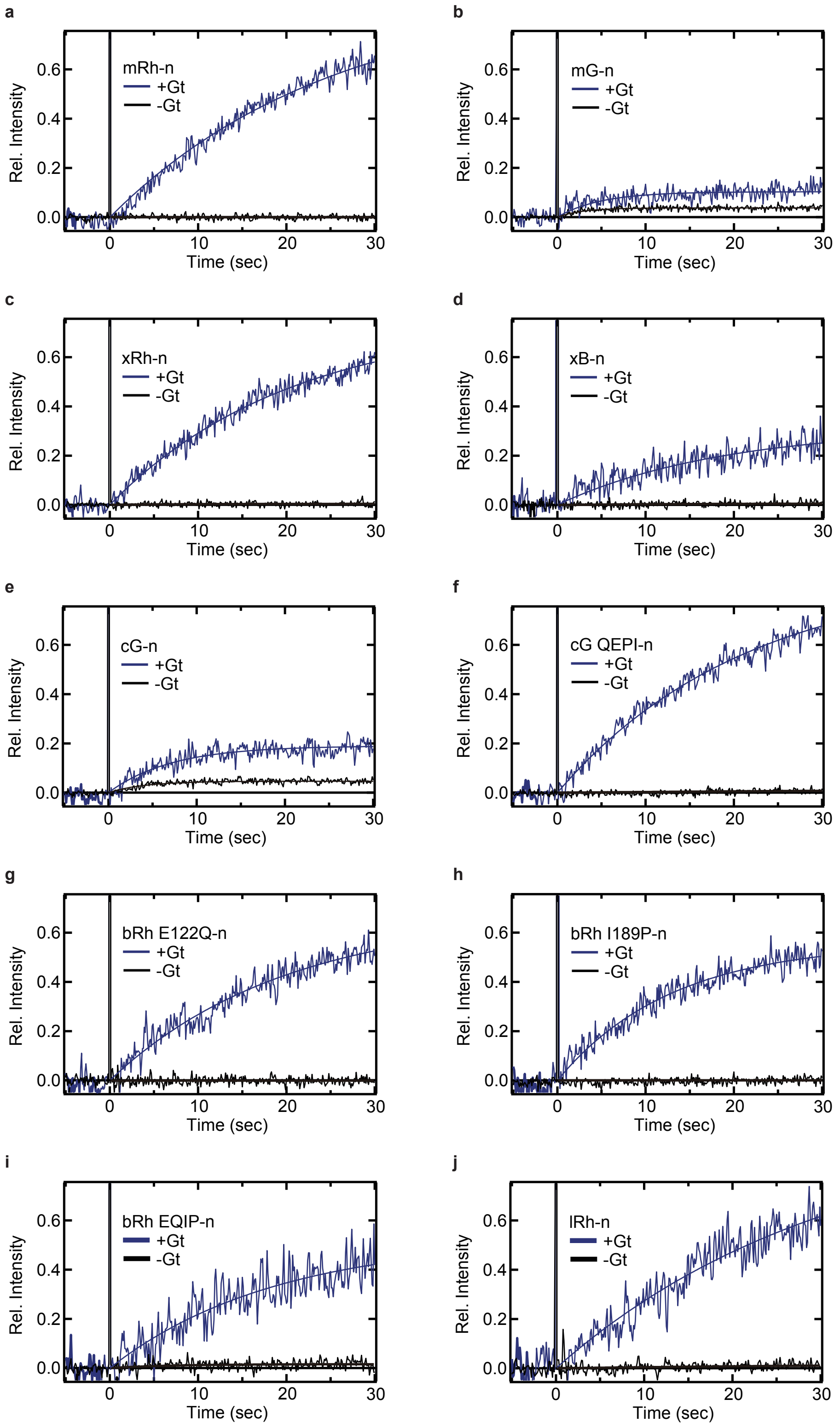

Supplementary Figure 4

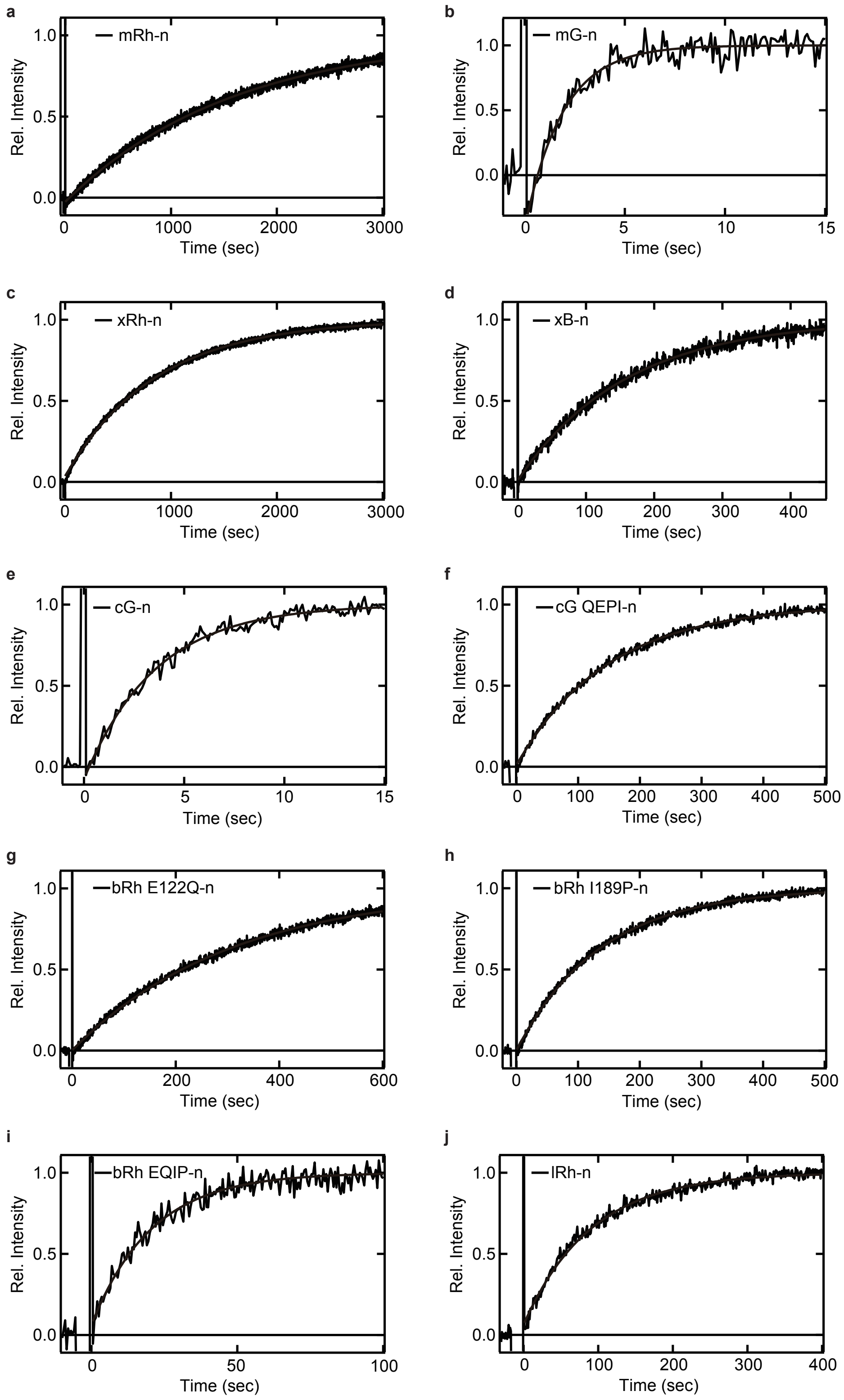

Supplementary Figure 5

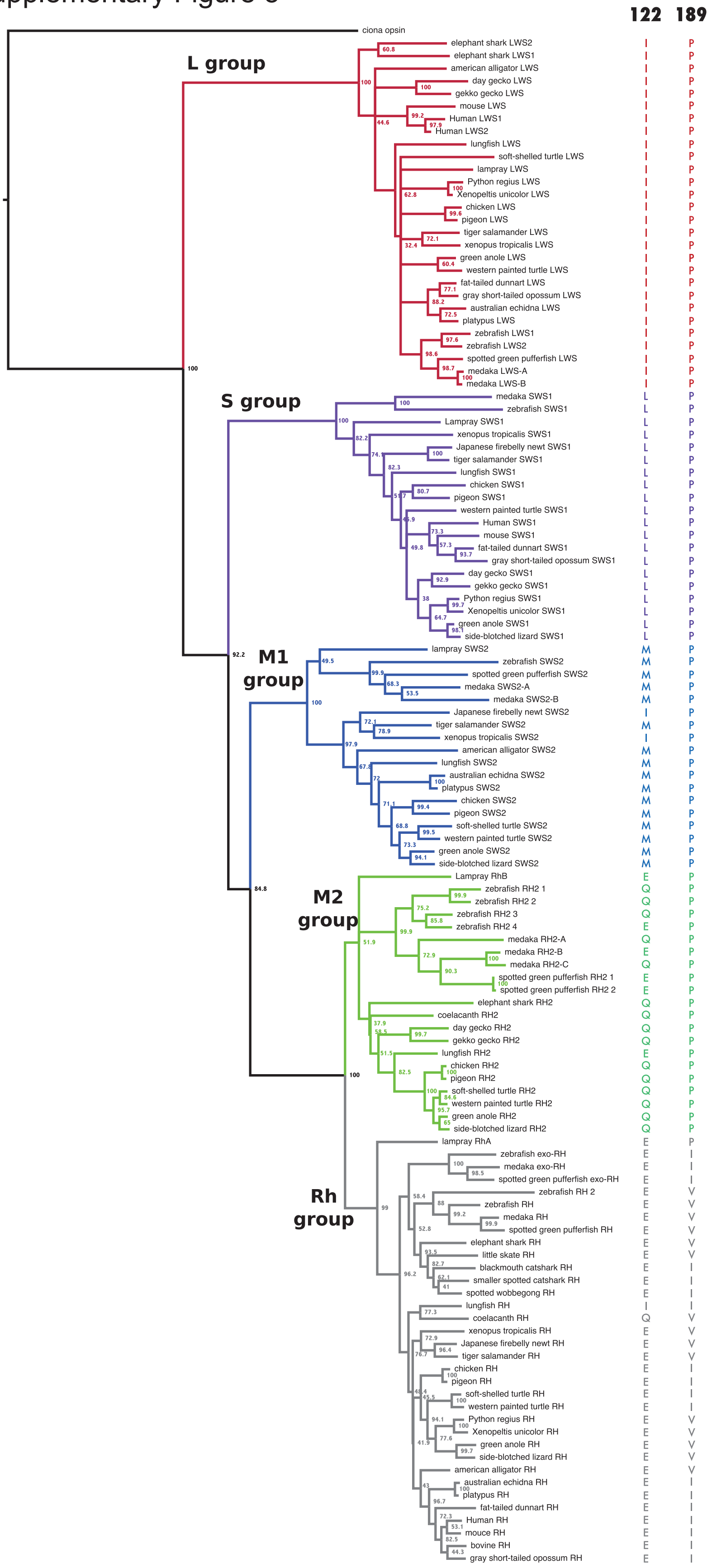

Supplement: Supplementary Information [file srep11081-s1.pdf]
